# Exploring Common Hub Genes in Thyroid Cancer and Hashimoto's Thyroiditis: Diagnostic Insights and Therapeutic Potential with Gefitinib

**DOI:** 10.2174/0113892029376349250819071649

**Published:** 2025-08-29

**Authors:** Ruqiong Sun, Bing Li, Fenjuan Xu, Juanfei Zhu

**Affiliations:** 1 Department of Endocrinology, Tongxiang First People's Hospital, Jiaochang Road 1918, 314500 Tongxiang, Zhejiang, P.R. China

**Keywords:** Thyroid cancer, hashimoto's thyroiditis, DEGs, hub genes, diagnosis

## Abstract

**Introduction:**

Thyroid Cancer (TC) is a prevalent endocrine malignancy with an increasing incidence worldwide, often associated with Hashimoto's Thyroiditis (HT), an autoimmune thyroid disorder. This study aimed to identify and validate key hub genes common to TC and HT and explore their diagnostic, prognostic, and therapeutic roles.

**Materials and Methods:**

Gene expression datasets for TC and HT were analyzed using bioinformatics tools to identify hub genes. In SW579 cells, Gefitinib treatment and siRNA-mediated knockdown of ALDH3A1 and DDX52 were performed, followed by RT-qPCR, Western blot, cell proliferation, colony formation, and wound healing assays.

**Results:**

After analyzing TC and HT datasets, we identified four common dysregulated hub genes: ALDH3A1, DDX52, RASA1, and SPATS2. RT-qPCR confirmed their significant upregulation in TC cell lines compared to normal controls (*p* < 0.001). ROC analysis demonstrated high diagnostic accuracy, with RASA1 and SPATS2 achieving AUC = 1. Gene expression validation using GSCA and HPA datasets corroborated these findings, and promoter hypomethylation analysis revealed regulatory mechanisms underlying their upregulation. Survival analyses associated elevated ALDH3A1 expression with poor overall survival. Functional assays in TC cells highlighted their oncogenic roles, with knockdown experiments showing reduced proliferation, migration, and colony formation. Immune correlation analyses revealed interactions with immune inhibitors and infiltrates, while miRNA profiling identified tumor-suppressive miRNAs targeting these genes. Drug prediction and molecular docking identified Gefitinib as a promising therapeutic, which effectively suppressed ALDH3A1 and DDX52 expression and oncogenic phenotypes in TC cells.

**Conclusion:**

This study offers comprehensive insights into the molecular underpinnings of TC progression, highlighting the diagnostic and therapeutic potential of these hub genes and their associated regulatory networks. These findings lay a foundation for developing novel therapeutic strategies targeting these genes in TC management.

## 1 INTRODUCTION

Thyroid Cancer (TC) is the most common endocrine malignancy and a significant public health concern, with its incidence steadily increasing over recent decades [1, 2]. TC comprises a heterogeneous group of tumors, primarily including Papillary Thyroid Carcinoma (PTC), Follicular Thyroid Carcinoma (FTC), Medullary Thyroid Carcinoma (MTC), and Anaplastic Thyroid Carcinoma (ATC) [3, 4]. Among these, PTC accounts for approximately 80–90% of cases and is generally associated with a favorable prognosis [4-6]. However, despite advancements in diagnostic techniques, surgical interventions, and targeted therapies, a subset of patients with TC still experiences recurrence, metastasis, or therapeutic resistance [7-9]. This highlights the urgent need for identifying novel molecular markers that can improve early diagnosis, predict patient outcomes, and offer new therapeutic targets. Hashimoto Thyroiditis (HT) is a chronic autoimmune thyroid disorder marked by lymphocytic infiltration, progressive destruction of thyroid follicles, and hypothyroidism [10, 11]. HT is one of the most common autoimmune diseases, predominantly affecting women. Its pathogenesis involves a complex interplay of genetic predisposition, environmental triggers, and immune dysregulation [12, 13]. Interestingly, a well-established but poorly understood association exists between HT and TC, particularly PTC [14]. Epidemiological studies reveal a higher prevalence of PTC in patients with HT, sparking interest in uncovering the underlying molecular mechanisms linking these two diseases [15]. The potential role of chronic inflammation, altered immune surveillance, and cytokine-mediated changes in the thyroid microenvironment is considered a critical contributor to this association [16]. However, the exact molecular pathways and key regulators driving this interplay remain largely elusive.

Hub genes, defined as highly connected nodes in biological networks, are central to maintaining cellular homeostasis and regulating disease pathogenesis [17-19]. These genes are often involved in critical biological processes such as cell proliferation, apoptosis, immune regulation, and metastasis [18, 20]. In cancer research, hub genes have emerged as powerful tools for understanding tumor biology, identifying biomarkers, and developing targeted therapies [21-23]. Similarly, in autoimmune diseases like HT, hub genes are implicated in immune signaling pathways, inflammation, and tissue remodeling [23-25]. Previous studies using transcriptomic analysis, Weighted Gene Co-expression Network Analysis (WGCNA), and Protein-protein Interaction (PPI) networks have identified disease-specific hub genes in TC and HT independently [26, 27]. However, limited research has focused on the intersection of these two conditions. Exploring the common hub genes between HT and TC is particularly intriguing, as it could reveal shared pathological mechanisms. For example, inflammation-driven carcinogenesis in HT could lead to the activation of oncogenic pathways, while immune-mediated tumor suppression may modify the tumor microenvironment in TC. Identifying common hub genes can offer insights into this dynamic crosstalk and reveal novel targets for diagnosis, prognosis, and therapeutic intervention.

Despite the growing understanding of TC and HT at the molecular level, the overlapping landscape of these diseases has not been thoroughly explored. The identification of shared hub genes has the potential to bridge the gap between autoimmune disorders and cancer research, offering dual benefits: improving the molecular understanding of HT-related thyroid carcinogenesis and identifying integrated biomarkers or therapeutic targets for patients with coexisting conditions. This study utilized advanced bioinformatics approaches and *in silico* validation [28] to identify common hub genes between HT and TC. Furthermore, these findings are experimentally validated using *in vitro* approaches to assess their diagnostic, prognostic, and therapeutic significance in TC.

## 2 MATERIALS AND METHODS

### 2.1 Data Collection and Processing

We retrieved gene expression datasets related to HT and TC from the GEO database, a publicly available repository curated by the NCBI that stores high-throughput gene expression data, including microarray and RNA sequencing datasets [29]. The datasets GSE6004 and GSE54958 for HT and GSE33630 and GSE53072 for TC were analyzed using the limma R package to identify Differentially Expressed Genes (DEGs). For DEG extraction, a threshold of |log2 fold change| > 1 and adjusted *p*-value < 0.05 was used. A Venn diagram was constructed to determine common DEGs (hub genes) across the datasets, focusing on the top 3000 DEGs from each dataset.

### 2.2 Cell Culture

A total of 12 thyroid cancer cell lines, BHP 5-16, 8505C, TPC-1, FTC-133, K1, BCPAP, SW579, WRO, CAL-62, OCUT-2, Nthy-ori 3-1, and ML-1 and six normal thyroid cell lines, Nthy-ori 3-1, HTori-3, Thy-3, TE 354.T, and TTA1 were purchased from American Type Culture Collection (ATCC). These cell lines were cultured in Dulbecco's Modified Eagle Medium (DMEM), supplemented with 10% Fetal Bovine Serum (FBS) and 1% penicillin-streptomycin. Additional supplements, such as sodium pyruvate, non-essential amino acids, or specific hormones like Thyrotropin (TSH) were also added. All cultures are maintained at 37°C in a humidified atmosphere containing 5% CO_2_.

### 2.3 RNA Extraction and Synthesis of the cDNA

RNA was extracted from the cell lines using an organic method [30, 31]. cDNA was synthesized using the RevertAid First Strand cDNA Synthesis Kit (Thermo Scientific) following the manufacturer’s instructions. RNA templates were first adjusted to a concentration of 0.5–1 μg per reaction. Each sample was then combined with 1 μl of oligo dT and incubated at 65°C for 5 minutes. Following this, a mixture containing 1 μl of primer, nuclease-free water up to 12 μl, 4 μl of 5X reaction buffer, 1 μl of Ribolock RNase inhibitor, 2 μl of 10 mM dNTP mix, and 1 μl of RevertAid M-MuLV RT was added. The samples were thoroughly mixed, briefly centrifuged, and then incubated for 60 minutes at 42°C, followed by 5 minutes at 70°C.

### 2.4 RT-qPCR Analysis

The RT-qPCR reaction mixture consisted of 10 µl of SensiFast Lo-ROX reagent (Bioline), 0.8 µl of a primer mixture containing forward and reverse primers, 1 µl of the cDNA sample, 0.1 µl of Taq polymerase, and 8.1 µl of distilled water, resulting in a total volume of 20 µl. The reactions were carried out using the QuantStudio 5 system (Thermo Fisher Scientific) according to the manufacturer’s instructions. Glyceraldehyde 3-phosphate Dehydrogenase (GAPDH) was used as the housekeeping or reference gene, and its expression was evaluated alongside all candidate genes.

The amplification results were computed using the following formula:

ΔΔCt=ΔCt[a target sample]−ΔCt[a reference sample]

The following primers were used for amplification purposes:

GAPDH-F 5'-ACCCACTCCTCCACCTTTGAC-3',

GAPDH-R 5'-CTGTTGCTGTAGCCAAATTCG-3'

ALDH3A1-F: 5'-CTCGTCATTGGCACCTGGAACT-3'

ALDH3A1-R: 5'-CTCGCCATGTTCTCACTCAGCT-3'

DDX52-F: 5'-GCTACATTTCAGCAACTTGACCAG-3'

DDX52-R: 5'-GATCCAGTTGGAGCAGAAGCCA-3'

RASA1-F: 5'-GGGACATCCAATAAACGCCTTCG-3'

RASA1-R: 5'-TTTGCTACTTGGACACTATTCAGG-3'

SPATS2-F: 5'- GTGTCTCTTGCACGGTATCGAG-3'

SPATS2-R: 5'-AAGCAACGCCACTTCTCGATCC-3'

### 2.5 Diagnostic Evaluation

Receiver Operating Characteristic (ROC) curve analysis was performed to assess the diagnostic potential of the hub genes. ROC curves and Area Under the Curve (AUC) values were calculated using the pROC R package.

### 2.6 Gene Set Enrichment Analysis (GSEA)

GSEA was conducted using the GSCA database, an integrative platform that provides multi-omics data and functional tools to analyze cancer genomics [32]. Normalized Enrichment Scores (NES) and adjusted *p*-values were used to confirm statistical significance.

### 2.7 Validation in Extended Cohorts

The expression of hub genes was validated using TC tissue datasets from the GSCA database [32] and the Human Protein Atlas (HPA) [33]. The mRNA expression levels were analyzed *via* the GSCA database across tumor and normal tissues, as well as different pathological stages. Immunohistochemistry (IHC) images from HPA were examined to validate protein-level expression.

### 2.8 Promoter Methylation and Correlation Analysis

Promoter methylation data for ALDH3A1, DDX52, RASA1, and SPATS2 were retrieved from the UALCAN database, an interactive web portal that provides access to publicly available cancer transcriptomic data, including TCGA datasets [34]. Correlations between promoter methylation levels and mRNA expression were analyzed using Pearson correlation coefficients *via* the GSCA database [32]. Survival analysis based on methylation levels was performed using Kaplan-Meier plots.

### 2.9 Genetic Alteration Analysis

Mutational and Copy Number Variation (CNV) data were analyzed using the cBioPortal and GSCA databases. These databases are platforms integrating genomic data from The Cancer Genome Atlas (TCGA) and other projects [35]. Mutation types, frequencies, and distributions were visualized, and CNV patterns were examined for heterozygous amplifications or deletions. Protein domain mapping was performed to identify functionally significant regions affected by mutations.

### 2.10 Prognostic Analysis

Overall Survival (OS) analysis was conducted using the Survival Genie [36], an online tool that integrates survival data from TCGA and GEO, and the GENT2 database [37], which aggregates expression profiles from various microarray datasets. Hazard Ratios (HR) and 95% Confidence Intervals (CI) were calculated to assess the prognostic significance of hub genes.

### 2.11 Correlation Analysis of Hub Genes with Immune Inhibitors

The correlation between the hub genes ALDH3A1, DDX52, RASA1, and SPATS2 and immune inhibitors in TC was evaluated using the TISIDB database [38]. This web-based portal integrates multiple datasets to analyze interactions between tumors and the immune system. Spearman’s rank correlation coefficients were calculated to assess the strength and significance of associations between hub genes and various immune inhibitors.

### 2.12 miRNA-mRNA Interaction Analysis

To identify miRNAs targeting hub genes, the TargetScanHuman database [39], a tool predicting miRNA binding sites based on sequence pairing and conservation, was used. Candidate miRNAs were selected based on high context++ scores and favorable site types. The expression levels of miRNAs identified (hsa-miR-4755-3p for ALDH3A1, hsa-miR-181b-5p for DDX52, hsa-miR-31-5p for RASA1, and hsa-miR-145-5p for SPATS2) were validated in TC cell lines and normal controls using RT-qPCR following the aforementioned protocol. U6 miRNA was used as a reference. Diagnostic potential was assessed using ROC curve analysis, and the Area Under the Curve (AUC) values was calculated to determine sensitivity and specificity.

### 2.13 PPI Network Construction and Gene Enrichment Analysis

Protein-protein Interaction (PPI) networks for the hub genes were constructed using the STRING [40] and GeneMANIA [41] databases. Venn analysis was performed to identify overlapping genes between the networks [42]. Functional enrichment analyses were conducted using the DAVID database to identify enriched biological processes, molecular functions, cellular components, and signaling pathways associated with these genes [43]. The results were visualized using bar and bubble plots.

### 2.14 Immune Infiltration Analysis

The relationship between hub gene expression and immune cell infiltration in TC was assessed using the GSCA database [32]. Correlation analysis was performed to determine associations between hub genes and various immune cell types, including Dendritic Cells (DCs), monocytes, regulatory T cells (Tregs), and more.

### 2.15 Drug Prediction and Molecular Docking Analysis

Drug sensitivity and resistance were analyzed using the GSCA database [32], with a focus on associations between hub genes and potential therapeutic agents. Gefitinib was identified as a candidate drug. Molecular docking studies were conducted to evaluate the binding affinities between Gefitinib and the hub gene-encoded proteins ALDH3A1 and DDX52. For this purpose, we employed the PubChem database to obtain the PDB structures of targeted drugs [44]. Then, we employed the ENSEMBLE database [45] to obtain FASTA sequences of the proteins encoded by hub genes. After that, we employed the SwissModel database [46] to model PDB structures of the proteins using FASTA sequences. In the end, we used the SeamDock tool [47] to conduct molecular docking, thereby forecasting the binding sites and binding strength between proteins and drugs.

### 2.16 Drug Preparation

Gefitinib (Thermo Fisher Scientific) was dissolved in DMSO to prepare a 10 mM stock solution, which was stored at -20°C for long-term use. Before treatment, the stock solution was diluted in the appropriate culture medium to achieve the desired working concentration. Based on a previously reported IC50 value of 2.89 ± 0.2 µM, this concentration was selected to investigate Gefitinib's inhibitory effects on ALDH3A1 and DDX52 in SW579 cells [48]. SW579 cells were seeded in 6-well plates at a density of 5 × 10^5^ cells per well and incubated overnight at 37°C with 5% CO_2_ to allow for cell attachment. The culture medium was then replaced with fresh medium containing 2.89 µM Gefitinib or an equal volume of DMSO for the control group. After 48 hours of treatment under standard culture conditions, cells were harvested for RT-qPCR to assess mRNA expression levels of ALDH3A1 and DDX52 at the mRNA level.

### 2.17 ALDH3A1 and DDX52 Knockdown in SW579 Cells

SW579 cells were seeded in 6-well plates at a density of 5 × 10^5^ cells per well and allowed to adhere overnight. Knockdown of ALDH3A1 and DDX52 was performed using gene-specific small interfering RNAs (siRNAs) and Lipofectamine™ RNAiMAX Transfection Reagent (Thermo Fisher Scientific) following the manufacturer’s protocol. Briefly, siRNA and Lipofectamine™ RNAiMAX were diluted in Opti-MEM™ Reduced Serum Medium (Thermo Fisher Scientific) and incubated at room temperature for 10 minutes to form transfection complexes. The complexes were added to the cells in complete medium and incubated for 48 hours under standard culture conditions (37°C, 5% CO_2_).

Total RNA was extracted from siRNA-transfected and control SW579 cells using the RNeasy Mini Kit (Qiagen) according to the manufacturer’s protocol. RT-qPCR was conducted following the mentioned protocol. Total protein was extracted from siRNA-transfected and control SW579 cells using RIPA Lysis Buffer (Thermo Fisher Scientific) supplemented with a protease inhibitor cocktail (Sigma-Aldrich). Protein concentrations were determined using the Pierce™ BCA Protein Assay Kit (Thermo Fisher Scientific). Equal amounts of protein (20 µg) were separated by SDS-PAGE and transferred onto PVDF membranes (Millipore). Membranes were blocked with 5% non-fat dry milk in TBS-T and incubated overnight at 4°C with primary antibodies against ALDH3A1, DDX52, and GAPDH (loading control). After washing, membranes were incubated with HRP-conjugated secondary antibodies. Bands were visualized using the ECL™ Detection Reagent (GE Healthcare) and quantified using ImageJ software.

### 2.18 Cell Proliferation Assay

Cell proliferation was evaluated using the Cell Counting Kit-8 (CCK-8, Dojindo Molecular Technologies). SW579 cells were seeded in 96-well plates at a density of 3 × 10^3^ cells per well and transfected with siRNAs as described above. After 48 hours, 10 µL of CCK-8 reagent was added to each well, followed by a 2-hour incubation at 37°C. Absorbance was measured at 450 nm using a microplate reader (BioTek).

### 2.19 Colony Formation Assay

Following siRNA transfection, SW579 cells were seeded in 6-well plates at a density of 500 cells per well and cultured for 10–14 days, with medium replacement every 3 days. Colonies were fixed with methanol and stained with a 0.5% crystal violet solution. Colonies containing ≥30 cells were counted under a microscope.

### 2.20 Wound Healing Assay

SW579 cells were transfected with siRNAs, seeded in 6-well plates, and grown to confluence. A uniform scratch was made using a sterile 200-µL pipette tip, and cells were washed with PBS to remove debris. The medium was replaced with serum-free DMEM, and wound closure was monitored at 0 and 24 hours using an inverted microscope. Images were analyzed using ImageJ software to calculate the percentage of wound closure.

### 2.21 Statistics

Statistical analysis was performed using GraphPad Prism (version 9.0, GraphPad Software, San Diego, CA, USA). Comparisons between two groups were performed using an unpaired Student’s t-test, and differences between multiple groups were analyzed using one-way Analysis of Variance (ANOVA) followed by Tukey’s post-hoc test for pairwise comparisons. For correlation analyses, Spearman’s rank correlation coefficient (rho) was calculated. ROC curve analysis was performed to evaluate the diagnostic performance. A p*-value < 0.05, p**-value < 0.01, and p***-value < 0.001 were considered statistically significant.

## 3 RESULTS

### 3.1 Identification and Validation of DEGs and Hub Genes in HT and TC

**Fig. (1):**
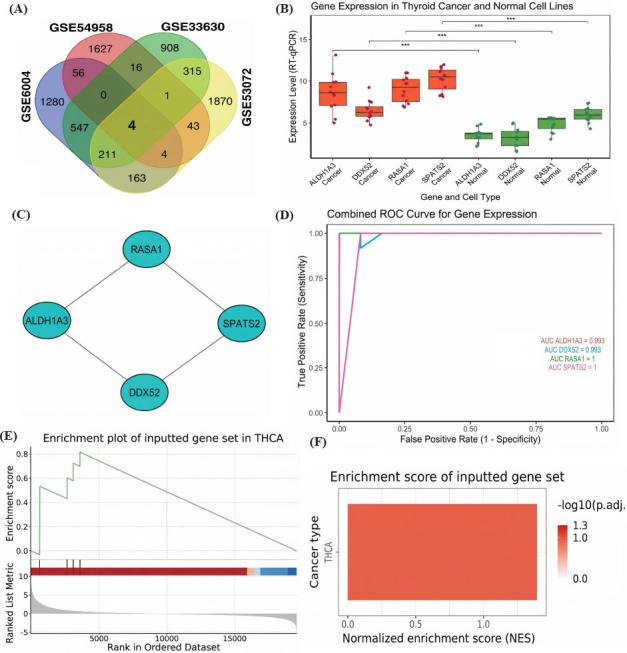
Identification and validation of common hub genes in HT and TC. (**A**) Venn diagram showing overlapping DEGs from the HT and TC datasets. (**B**) A PPI network of the ALDH1A3, DDX52, RASA1, and SPATS2 hub genes. (**C**) RT-qPCR analysis of hub gene expression in TC and normal control cell lines. (**D**) ROC curves demonstrating diagnostic performance of the hub genes. (**E-F**) GSEA results illustrating significant enrichment of hub genes in TC. *P*-value*** < 0.001.

In the first part of our study, we identified and validated key common hub genes involved in TC and HT. For this purpose, two HT datasets (GSE6004 and GSE54958) and two TC datasets (GSE33630 and GSE53072) were retrieved from the GEO database and analyzed using limma package. Fig. (**1A**) shows a Venn diagram of the top 3000 DEGs from each dataset. This analysis revealed four common hub genes including ALDH1A3, DDX52, RASA1, and SPATS2, that were consistently dysregulated across both HT and TC datasets (Fig. **1A**-**B**). Next, the expressions of hub genes were measured using RT-qPCR. Fig. (**1C**) presents the results of the RT-qPCR expression analysis, which we performed on 12 TC cell lines and 6 normal control cell lines. The expression levels of ALDH1A3, DDX52, RASA1, and SPATS2 were significantly up-regulated (*p* < 0.001) in TC cell lines as compared to normal control cell lines. Based on the expression data, ROC analysis of the hub genes was conducted to evaluate their diagnostic powers. Fig. (**1D**) illustrates the ROC curves for these genes, where Area Under the Curve (AUC) approached 1 for all genes, with RASA1 and SPATS2 achieving perfect diagnostic accuracy (AUC = 1), indicating their strong ability to

distinguish between TC and normal individuals (Fig. **1D**). Fig. (**1E**-**F**) shows the results of the GSEA, which we conducted using the GSCA database. In Fig. (**1E**), the enrichment plot indicated a significant enrichment of our hub gene set in TC. In Fig. (**1F**), the Normalized Enrichment Score (NES) and the log10-adjusted *p*-value confirmed that this enrichment was statistically significant, further emphasizing the importance of these genes in TC pathogenesis.

### 3.2 Validation of Hub Gene Expression using Extended TC Cohorts

**Fig. (2):**
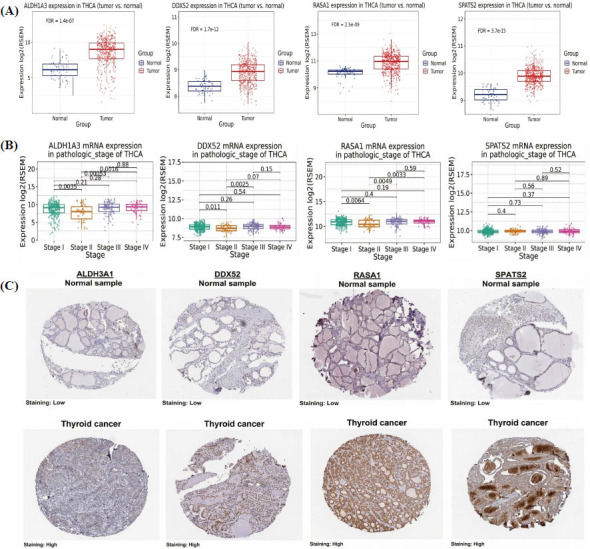
Validation of hub gene expression in TC cohorts. (**A**) mRNA expression levels of ALDH3A1, DDX52, RASA1, and SPATS2 in TC tissues *versus* normal tissues *via* the GSCA database. (**B**) Stage-wise mRNA expression levels of the hub genes *via* the GSCA database. (**C**) Immunohistochemistry images from the HPA database depicting protein expression of hub genes in TC and normal thyroid tissues. *P*-value < 0.05.

We validated the mRNA and protein expression levels of hub genes ALDH3A1, DDX52, RASA1, and SPATS2 in TC using the GSCA platform and the HP database. The mRNA expression analysis revealed a significant (*p* < 0.001) upregulation of all four hub genes in tumor tissues compared to normal tissues (Fig. **2A**). Stage-wise analysis >revealed that ALDH3A1 and DDX52 exhibited increasing expression with advancing pathological stages, whereas RASA1 demonstrated elevated expression in later stages, and SPATS2 displayed relatively stable levels across stages (Fig. **2B**). Protein-level validation using immunohistochemistry images from the HPA revealed low expression of these genes in normal thyroid tissues, whereas their expression was markedly increased in TC tissues, mirroring the mRNA findings (Fig. **2C**).

### 3.3 Promoter Methylation Analysis and its Association with Hub Gene Expression and Survival in TC

**Fig. (3):**
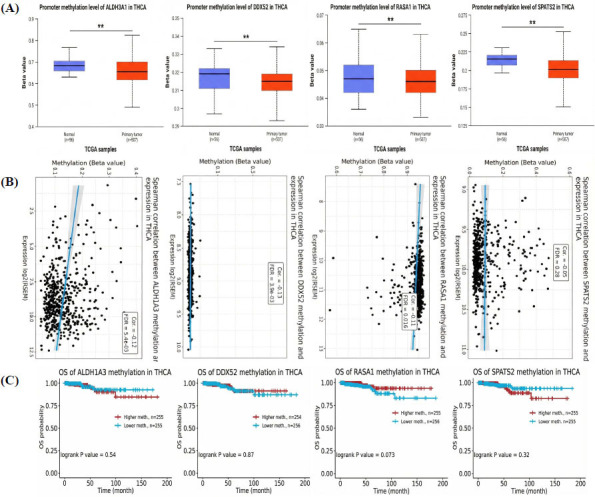
Promoter methylation and survival analysis of hub genes in TC. (**A**) Promoter methylation levels of hub genes in tumor and normal tissues. (**B**) Correlation between promoter methylation and mRNA expression of the hub genes. (**C**) Kaplan-Meier survival analysis based on promoter methylation levels. *P*-value** < 0.01.

Additionally, this study analyzed the promoter methylation levels and survival associations of hub genes (ALDH3A1, DDX52, RASA1, and SPATS2) in TC using the UALCAN and GSCA platforms. Promoter methylation analysis *via* the UALCAN database revealed significantly lower methylation levels of all four genes in primary tumor samples compared to normal tissues, indicating potential hypomethylation-driven upregulation (Fig. **3A**). Correlation analysis *via* the GSCA database demonstrated a strong negative relationship between promoter methylation levels and mRNA expression for ALDH3A1 (Cor = -0.57, FDR < 0.05), suggesting methylation as a key regulatory mechanism. However, no significant correlations were observed for DDX52, RASA1, or SPATS2 (Fig. **3B**). Kaplan-Meier survival analysis showed no statistically significant differences in Overall Survival (OS) between patients with higher and lower methylation levels of these genes, with log-rank *p*-values of 0.54, 0.87, 0.073, and 0.32 for ALDH3A1, DDX52, RASA1, and SPATS2, respectively (Fig. **3C**).

### 3.4 Genetic Alteration Analysis of Hub Genes in TC

**Fig. (4):**
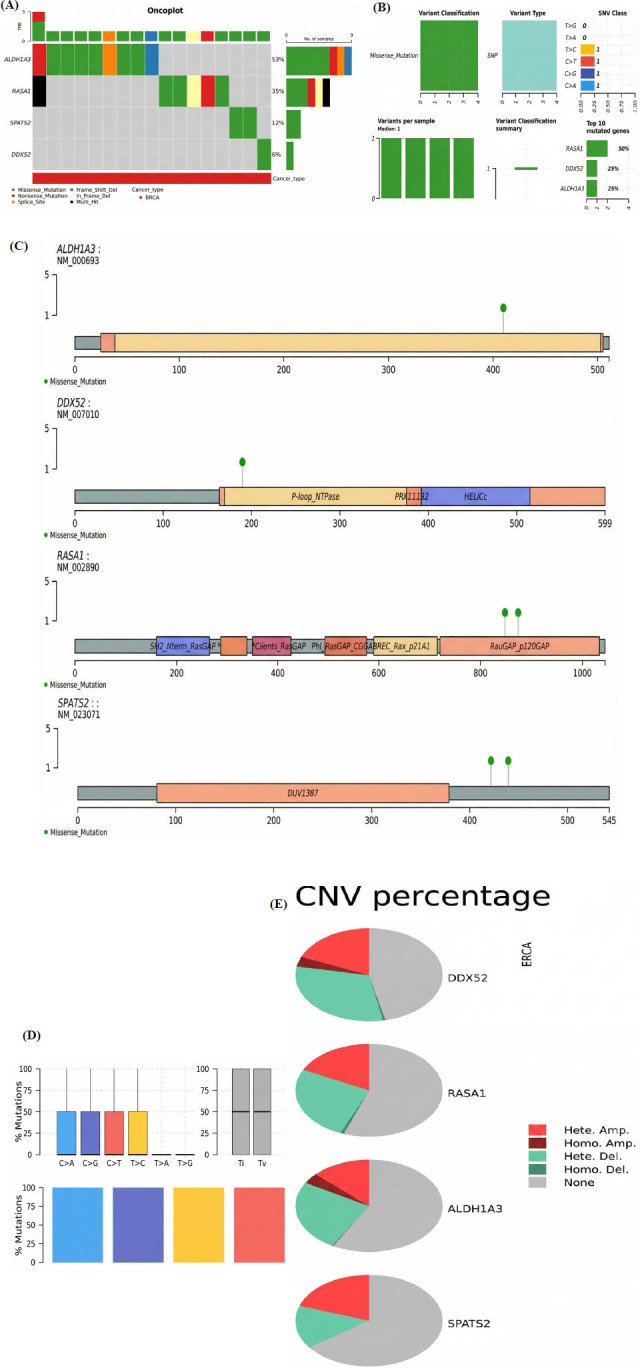
Genetic alterations analysis of hub genes in TC. (**A**) Mutation frequency of hub genes. (**B**) Distribution of mutation types across the hub genes. (**C**) Mapping of mutations to protein domains. (**D**) Transition and transversion events observed in mutations. (**E**) CNV analysis indicating heterozygous amplification in hub genes. *P*-value < 0.05.

We conducted mutational and CNV analysis for the hub genes ALDH3A1, DDX52, RASA1, and SPATS2 using the cBioPortal and GSCA databases. Mutation analysis revealed that ALDH3A1 had the highest mutation frequency (53%), followed by RASA1 (35%), SPATS2 (12%), and DDX52 (6%) in BRCA samples, with missense mutations being the predominant variant type (Fig. **4A**-**C**). Most mutations were classified as single-nucleotide polymorphisms (SNPs), with transition (Ti) and transversion (Tv) events showing balanced proportions (Fig. **4D**). Protein domain mapping highlighted the locations of key mutations, particularly in functionally significant regions of ALDH3A1, DDX52, and RASA1 (Fig. **4C**). CNV analysis indicated that LDH3A1, DDX52, RASA1, and SPATS2 primarily exhibited heterozygous amplification, reflecting varying genomic alterations (Fig. **4E**).

### 3.5 Prognostic Analysis of Hub Genes in TC

**Fig. (5):**
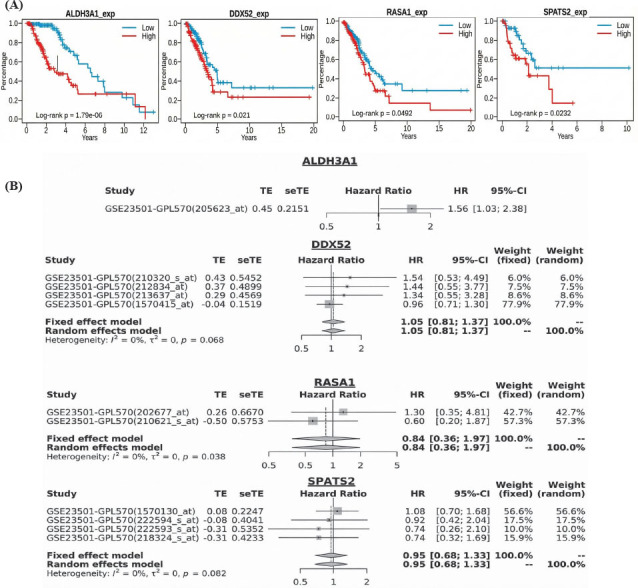
Prognostic value of hub genes in TC. (**A**) Kaplan-Meier survival curves showing overall survival association with hub gene expression. (**B**) Meta-analysis revealing hazard ratios for hub genes in TC prognosis. *P*-value < 0.05.

This study conducted a survival analysis of hub genes (ALDH3A1, DDX52, RASA1, and SPATS2) using Survival Genie and GENT2 meta-analysis tools. KM survival analysis revealed that high expression levels of these genes were significantly associated with poor overall survival in patients, with log-rank *p*-values of 1.79 × 10^-6^, 0.021, 0.0492, and 0.0232, respectively (Fig. **5A**). Patients with elevated expression of these genes exhibited shorter survival durations, highlighting their potential prognostic value (Fig. **5A**). Meta-analysis further revealed that ALDH3A1 had a significant Hazard Ratio (HR) of 1.56 (95% CI: 1.03–2.38), indicating its strong association with increased mortality risk, while DDX52, RASA1, and SPATS2 showed hazard ratios of 1.05 (95% CI: 0.81–1.37), 0.84 (95% CI: 0.36–1.97), and 0.95 (95% CI: 0.68–1.33), respectively, with no significant associations observed (Fig. **5B**). These results suggest that ALDH3A1 is a robust prognostic marker, while the roles of DDX52, RASA1, and SPATS2 in predicting survival outcomes may require further validation.

### 3.6 Correlations of Hub Genes with Immune Inhibitors in TC

**Fig. (6):**
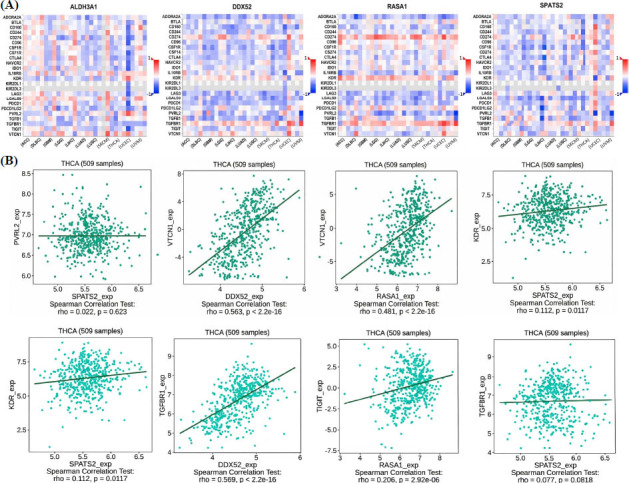
Correlation of hub genes with immune inhibitors in TC. (**A**) Heatmap illustrating correlations between hub genes and immune inhibitors in pan-cancer. (**B**) Scatter plots highlighting significant correlations for ALDH3A1, DDX52, RASA1, and SPATS2 in TC. *P*-value < 0.05.

Additionally, we performed correlation analysis for the hub genes ALDH3A1, DDX52, RASA1, and SPATS2 with various immune inhibitors in TC using the TISIDB database. Heatmap analysis revealed positive correlations of these genes in association with various immune inhibitors in TC samples (Fig. **6A**). For instance, a significant positive correlation was observed between DDX52 and VTCN1 (rho = 0.563, *p* < 2.2 × 10^-16^) as well as DDX52 and TGFBR1 (rho = 0.569, *p* < 2.2 × 10^-16^). Similarly, RASA1 exhibited a positive correlation with VTCN1 [rho = 0.481, *p* < 2.2 × 10^-16^) and TIGIT (rho = 0.206, *p* = 2.92 × 10^-6^). In contrast, SPATS2 displayed weaker correlations, with a modest association observed with KDR (rho = 0.112, *p* = 0.0117) (Fig. **6B**).

### 3.7 miRNA-mRNA Analysis of Hub Genes in TC

**Fig. (7):**
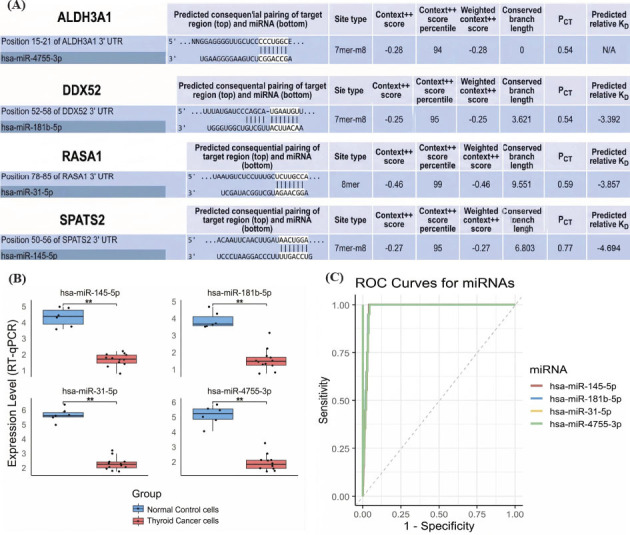
miRNA-mRNA regulatory network of hub genes. (**A**) TargetScanHuman predictions for miRNAs targeting hub genes. (**B**) RT-qPCR analysis of miRNA expression in TC cell lines. (**C**) ROC curves demonstrating the diagnostic performance of miRNAs. *P*-value** < 0.01.

We investigated miRNAs targeting the hub genes ALDH3A1, DDX52, RASA1, and SPATS2 using the TargetScanHuman database, followed by expression analysis and ROC curve evaluation. TargetScanHuman analysis identified hsa-miR-4755-3p as a potential regulator of ALDH3A1, hsa-miR-181b-5p for DDX52, hsa-miR-31-5p for RASA1, and hsa-miR-145-5p for SPATS2, based on predicted consequential pairing, site types, and favorable context++ scores (Fig. **7A**). Expression analysis *via* RT-qPCR revealed significant downregulation of hsa-miR-145-5p, hsa-miR-181b-5p, hsa-miR-31-5p, and hsa-miR-4755-3p in TC cell lines compared to normal control cell lines (*p* < 0.01 for all comparisons), suggesting their potential tumor-suppressive roles (Fig. **7B**). ROC curve analysis demonstrated high sensitivity and specificity for these miRNAs in distinguishing TC samples from controls, with hsa-miR-145-5p, hsa-miR-181b-5p, hsa-miR-31-5p, and hsa-miR-4755-3p yielding AUC values close to 1.0, further validating their diagnostic potential (Fig. **7C**).

### 3.8 PPI Network Construction and Gene Enrichment Analysis of the Hub Genes

**Fig. (8):**
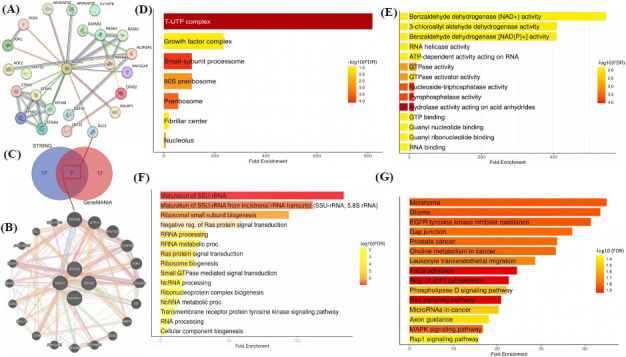
PPI network and enrichment analysis of hub genes. (**A-B**) PPI networks constructed using STRING and GeneMANIA databases. (**C**) Venn diagram showing overlapping genes between the two networks. (**D-G**) Functional enrichment analysis showing cellular components, molecular functions, biological processes, and pathways associated with hub genes. *P*-value < 0.05.

We constructed the PPI networks of the hub genes *via* the STRING and GeneMANIA databases (Fig. **8A**-**B**). Furthermore, Venn analysis revealed seven overlapping genes between the two networks, emphasizing their shared importance (Fig. **8C**). Functional enrichment analysis of the common genes using DAVID provided insights into their roles in biological processes, molecular functions, and pathways. Cellular component analysis (Fig. **8D**) highlighted their association with structures like the “T-UTP complex, 90S preribosome, and fibrillar center, emphasizing their roles in ribosomal assembly and nucleolar functions.” Molecular function enrichment revealed activities such as “RNA binding, GTPase activity, and benzaldehyde dehydrogenase activity, reflecting their roles in RNA metabolism and enzymatic functions” ((Fig. **8E**). Biological process enrichment showed significant involvement in “ribosomal RNA (Rrna) processing, maturation of SSU-rRNA, and Ras protein signal transduction, suggesting roles in ribosome biogenesis and oncogenic signaling” (Fig. **8F**). Pathway analysis demonstrated the involvement of these genes in cancer-related pathways, including “melanoma, glioma, EGFR tyrosine kinase inhibitor resistance, Ras signaling, and MAPK signaling” (Fig. **8G**).

### 3.9 Immune Infiltration, Drug Prediction, Molecular Docking Analyses, and Treatment of SW579 with Gefitinib to Inhibit the Expression of Hub Genes

**Fig. (9):**
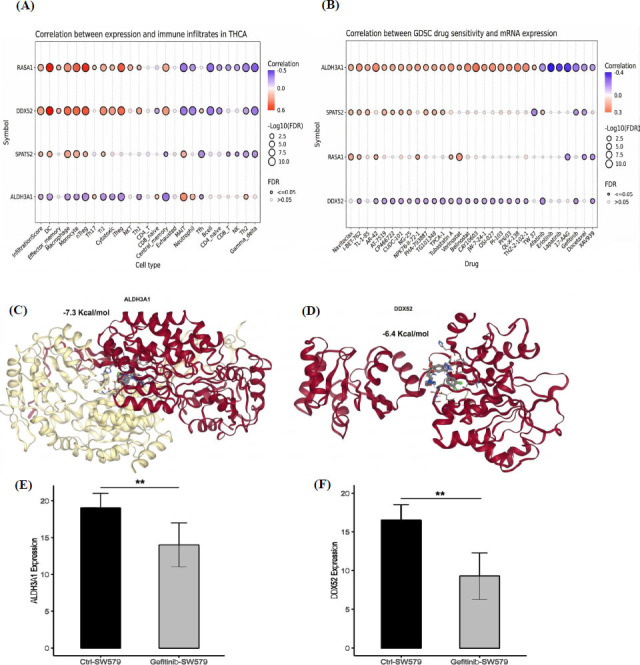
Immune infiltration, drug prediction, molecular docking, and Gefitinib validation in TC. (**A**) Immune infiltration analysis correlating hub gene expression with immune cell types. (**B**) Drug prediction analysis showing resistance and sensitivity profiles for hub genes. (**C-D**) Molecular docking results for Gefitinib binding to ALDH3A1 and DDX52. (**E-F**) RT-qPCR validation of ALDH3A1 and DDX52 expression after Gefitinib treatment in SW579 cells. *P*-value** < 0.01.

This study further conducted immune infiltration, drug prediction, molecular docking, and validation analyses of hub genes in TC. Immune infiltration analysis using the GSCA database revealed distinct correlations between the expression of these hub genes and various immune cell types. RASA1 showed a strong positive correlation with immune infiltrates, particularly with DC, monocytes, and Tr1 cells (Fig. **9A**). DDX52 exhibited significant positive correlations nTreg and Th17 cells (Fig. **9A**). SPATS2 displayed a strong negative association with infiltrating B cells (Fig. **9A**). Meanwhile, ALDH3A1 demonstrated unique negative correlations with monocytes and nTreg cells (Fig. **9A**). Drug prediction analysis was conducted to evaluate the association of these hub genes with drug sensitivity using the GSCA database. Although, hub genes were found causing resistance to various drugs, including AR-42 and Novitoclas (Fig. **9B**), Gefitinib was identified as a promising drug candidate for TC treatment with respect to hub genes all these genes showed the strongest correlations with gefitinib sensitivity, with ALDH3A1 and DDX52 having a more pronounced association with this drug (Fig. **9B**).

To further validate the effect of Gefitinib, molecular docking was performed to assess the interaction between Gefitinib and the target proteins (ALDH3A1 and DDX52). The docking results showed strong binding affinities for Gefitinib with ALDH3A1 (-7.3 kcal/mol) and DDX52 (-6.4 kcal/mol), indicating favorable interactions and potential efficacy in targeting these proteins (Fig. **9C**-**D**). Experimental validation was then carried out by applying gefitinib to the TC cell line SW579, followed by RT-qPCR analysis to assess the expression levels of ALDH3A1 and DDX52. The results demonstrated significant downregulation of both ALDH3A1 and DDX52 in SW579 cells after gefitinib treatment (*p* < 0.01), further supporting its therapeutic potential in TC (Fig. **9E**-**F**).

### 3.10 Functional Roles of ALDH3A1 and DDX52 in Thyroid Cancer Progression and Proposed Mechanistic Model

In the final part of the study, we investigated the functional roles of ALDH3A1 and DDX52 in TC by performing gene knockdown experiments in SW579 cells. Knockdown of ALDH3A1 and DDX52 was validated at both the mRNA and protein levels using RT-qPCR and Western blotting, respectively. The results demonstrated significant downregulation of ALDH3A1 and DDX52 expression in si-ALDH3A1-SW579 and si-DDX52-SW579 cells compared to control SW579 cells (*p* < 0.01) Fig. (**10A**-**C**).

**Fig. (10):**
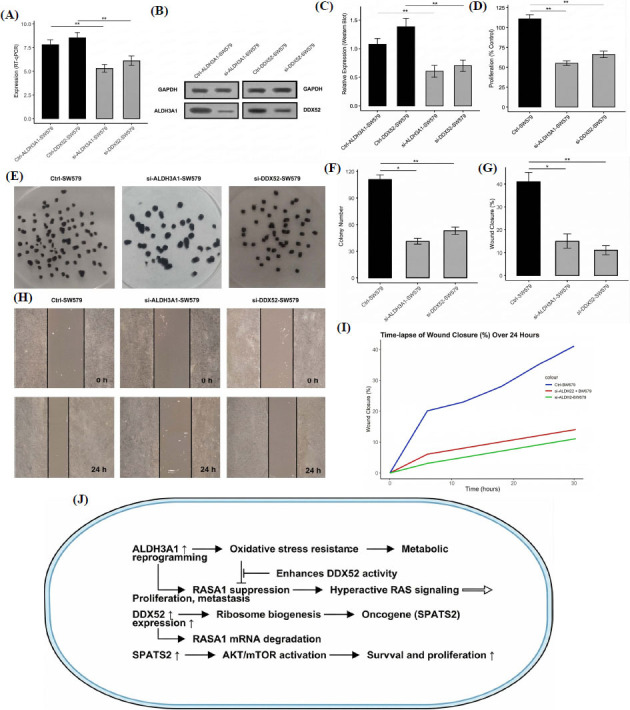
Functional validation and proposed model of hub gene-mediated TC progression. (**A-B**) RT-qPCR and Western blot validation of ALDH3A1 and DDX52 knockdown. (**C-I**) Functional assays showing reduced proliferation, colony formation, and migration after knockdown. (**J**) Proposed model illustrating the roles of ALDH3A1, DDX52, RASA1, and SPATS2 in TC pathogenesis through oxidative stress resistance, ribosome biogenesis, oncogenic signaling, and RAS pathway activation. *P*-value** < 0.01.

Functional assays revealed significant suppression of oncogenic phenotypes following ALDH3A1 and DDX52 knockdown. Specifically, cell proliferation was significantly reduced in si-ALDH3A1-SW579 and si-DDX52-SW579 cells compared to controls (*p* < 0.01) (Fig. **10D**). Colony formation assays showed a significant decrease in colony number after knockdown of these genes (*p* < 0.01) (Fig. **10E** and **F**). Moreover, wound healing assays indicated that cell migration was significantly impaired in si-ALDH3A1-SW579 and si-DDX52-SW579 cells, with slower wound closure compared to control cells (*p* < 0.01) (Fig. **10G**, **H**, and **I**).

Based on these experimental findings, ultimately, we proposed a model that explains how the upregulation of ALDH3A1, DDX52, RASA1, and SPATS2 contributes to TC progression (Fig. **10J**). Upregulated ALDH3A1 promotes oxidative stress resistance and metabolic reprogramming, enhancing the activity of DDX52 (Fig. **10J**). Upregulated DDX52, in turn, supports ribosome biogenesis and oncogene (*e.g*., SPATS2) expression (Fig. **10J**). Upregulated SPATS2 activates the AKT/mTOR signaling pathway, driving cell survival and proliferation (Fig. **10J**). Additionally, ALDH3A1-mediated suppression of RASA1 leads to hyperactivation of RAS signaling, further promoting proliferation and metastasis (Fig. **10J**).

## 4 DISCUSSION

Hashimoto’s Thyroiditis (HT), an autoimmune disorder characterized by chronic inflammation and thyroid dysfunction, has been linked to an increased risk of developing Thyroid Cancer (TC), particularly Papillary Thyroid Carcinoma (PTC) [49-52]. However, the molecular mechanisms connecting HT and TC remain poorly understood. Identifying shared molecular drivers could significantly enhance the understanding of the pathogenesis of these diseases and provide valuable diagnostic and therapeutic insights. While previous studies have independently explored gene expression changes in HT and TC, few have systematically examined overlapping dysregulated genes or their functional roles [50, 53]. This study aimed to address this gap by integrating transcriptomic data from HT and TC datasets, identifying common DEGs and hub genes, and validating their functional and therapeutic potential.

Our analysis identified four hub genes, including ALDH3A1, DDX52, RASA1, and SPATS2, that were consistently dysregulated in the HT and TC datasets. Expression validation revealed significant upregulation of these genes in TC cell lines and tissues compared to normal controls. These findings are consistent with prior studies implicating similar molecular pathways in thyroid carcinogenesis, including oxidative stress resistance, ribosome biogenesis, and oncogenic signaling [54-56]. Importantly, this study provides new insights into how these genes contribute to TC progression and their potential as therapeutic targets.

ALDH3A1, identified as one of the key hub genes, plays a crucial role in promoting resistance to oxidative stress and facilitating metabolic reprogramming [57]. Previous research has highlighted the role of aldehyde dehydrogenases in various cancers [58, 59], where they facilitate resistance to oxidative damage and chemotherapeutics [60]. However, our findings extend this understanding by demonstrating that ALDH3A1 suppresses RASA1, leading to the hyperactivation of RAS signaling —a key driver of cell proliferation and metastasis [61, 62]. This novel mechanistic insight emphasizes the dual role of ALDH3A1 in metabolic adaptation and oncogenic signaling in TC. Similarly, DDX52, a gene traditionally recognized for its role in ribosomal RNA processing [63, 64], was shown in our study to be upregulated in TC, supporting ribosome biogenesis and the expression of oncogenes such as SPATS2. While previous reports have linked ribosome biogenesis to cancer, our data provide the first evidence of DDX52’s involvement in TC, further highlighting its potential as a therapeutic target [65].

RASA1, a known regulator of RAS signaling, demonstrated elevated expression in TC and was associated with hyperactive Ras-mediated oncogenic pathways [66]. While earlier studies have implicated RASA1 dysregulation in various malignancies, our data provide new insights into its role in TC, where its upregulation appears to contribute to enhanced cell proliferation and migration [67, 68]. SPATS2, the fourth hub gene identified, exhibited consistent upregulation in TC and was shown to activate the AKT/mTOR signaling pathway, a key driver of cell survival and proliferation [69]. Although SPATS2 has been associated with oncogenic pathways in other cancers, its role in thyroid cancer remains underexplored, making this finding significant [70, 71].

Our findings also shed light on the epigenetic regulation of these hub genes. Promoter methylation analysis revealed hypomethylation-driven upregulation of ALDH3A1, whereas no significant correlation was observed between promoter methylation and gene expression for the other hub genes. These results suggest that different regulatory mechanisms may underlie the expression of these genes, emphasizing the need for further investigation into their epigenetic control. Functional assays further validated the oncogenic roles of ALDH3A1 and DDX52. Knockdown experiments in SW579 TC cells demonstrated significant suppression of cell proliferation, colony formation, and migration, confirming their critical roles in driving TC progression. Comparing these results with existing studies highlights both consistencies and novelties. While prior research has established the involvement of oxidative stress, ribosome biogenesis, and oncogenic signaling in cancer, our study uniquely integrates transcriptomic, epigenetic, and functional analyses to provide a comprehensive understanding of these processes in TC [72-74].

In recent years, the application of Epidermal Growth Factor Receptor (EGFR) Tyrosine Kinase Inhibitors (EGFR-TKIs), such as Gefitinib, has garnered significant attention as a potential therapeutic approach in TC [75, 76]. EGFR overexpression is commonly observed in various cancers, including TC [76, 77], and is often associated with poor prognosis and resistance to conventional therapies. Gefitinib, an EGFR-TKI, inhibits EGFR signaling, which plays a crucial role in tumor growth and progression [78, 79]. Other EGFR-TKIs, such as Erlotinib, Afatinib, and Osimertinib, have also been explored for their potential in TC treatment. Erlotinib, like Gefitinib, inhibits EGFR signaling by blocking its tyrosine kinase activity, and has been shown to reduce tumor growth in various cancers, including head and neck squamous cell carcinoma [80]. However, its effectiveness in TC remains under investigation. Afatinib, a second-generation EGFR-TKI, irreversibly binds to the EGFR receptor and targets both wild-type and mutant EGFR, showing promise in the treatment of EGFR-mutated cancers such as Non-small Cell Lung Cancer (NSCLC0 [81, 82]. Osimertinib, a third-generation EGFR-TKI, specifically targets both EGFR sensitizing mutations and the T790M resistance mutation, and has demonstrated superior efficacy in NSCLC [83], but its potential in TC is still being studied. Despite these advancements, resistance to EGFR-TKIs remains a significant challenge, emphasizing the need for combination therapies and personalized treatment strategies. In the present study, we inhibited ALDH3A1 and DDX52 using Gefitinib, which are implicated in the dysregulation of the RAS signaling network. The findings provide strong evidence for Gefitinib’s role in modulating the expression of ALDH3A1 and DDX52, contributing to the inhibition of the RAS pathway. Given the shared involvement of these genes in both HT and TC, the demonstrated sensitivity of ALDH3A1 and DDX52 to Gefitinib suggests that it could potentially be repurposed not only for TC treatment but also for HT management, especially in patients at high risk of developing TC. The upregulation of these genes in both conditions suggests that Gefitinib could prevent or reduce TC development in HT patients by targeting these common molecular pathways. However, further research is needed to explore whether Gefitinib or other inhibitors targeting ALDH3A1 and DDX52 can effectively intervene in the progression of HT and prevent TC. This dual therapeutic potential of Gefitinib would enhance its clinical relevance, providing valuable insights into managing both HT and TC through RAS pathway inhibition.

## STUDY LIMITATIONS

Although this study offers valuable insights into the role of hub genes in TC progression, there are a few limitations to consider. Firstly, due to the lack of HT cell lines, the analyses were conducted solely using TC cell lines. This limitation may impact the diagnostic potential of these genes in distinguishing TC patients from healthy individuals. Therefore, to better assess the diagnostic utility of these hub genes in distinguishing between HT normal individuals, clinical sample-based studies are needed, which could provide more comprehensive insights into their potential as diagnostic biomarkers for both conditions. Furthermore, while the study provides promising data on gene expression, genetic alterations, and functional assays, the long-term effects of hub gene manipulation in *in vivo* models are yet to be explored. Additionally, the findings related to promoter methylation and genetic alterations require further validation in larger, independent cohorts to confirm their robustness. Lastly, while the proposed mechanistic model provides insight into the role of ALDH3A1, DDX52, RASA1, and SPATS2 in TC and HT progression, additional studies are needed to validate the interactions and confirm their clinical relevance in therapeutic settings.

## CONCLUSION

This study identified four hub genes, including ALDH3A1, DDX52, RASA1, and SPATS2, that were dysregulated in both HT and thyroid cancer TC, providing insights into the shared molecular mechanisms between these conditions. Our analysis demonstrates the critical roles these genes play in TC progression, involving oxidative stress resistance, ribosome biogenesis, and oncogenic signaling pathways. Moreover, we explored the potential therapeutic use of Gefitinib, an EGFR inhibitor, which demonstrated the ability to downregulate hub genes, suggesting its repurposing for both TC and HT management. Our findings also revealed epigenetic regulation of these genes. Despite promising results, the study has limitations, including the absence of HT cell lines in functional assays and the need for *in vivo* validation. Future studies incorporating clinical samples, long-term effects, and larger cohorts are essential to confirm the clinical relevance of these genes as diagnostic biomarkers and therapeutic targets for both HT and TC. In conclusion, this study provides a comprehensive framework for understanding the molecular links between HT and TC and identifies potential therapeutic targets for clinical application.

## Data Availability

The URLs of all the publicly available analyzed datasets have been provided in the methodology section. Any additional information or specific dataset requests can be made from the corresponding author.

## LIST OF ABBREVIATIONS

ANOVA: Analysis of Variance
ATC: Anaplastic Thyroid Carcinoma
ATCC: American Type Culture Collection
AUC: Area Under the Curve
CI: Confidence Intervals
CNV: Copy Number Variation
DCs: Dendritic Cells
DEGs: Differentially Expressed Genes
DMEM: Dulbecco's Modified Eagle Medium
EGFR: Epidermal Growth Factor Receptor
FBS: Fetal Bovine Serum
FTC: Follicular Thyroid Carcinoma
GAPDH: Glyceraldehyde 3-phosphate Dehydrogenase
HPA: Human Protein Atlas
HR: Hazard Ratios
HT: Hashimoto's Thyroiditis
IHC: Immunohistochemistry
MTC: Medullary Thyroid Carcinoma
NES: Normalized Enrichment Scores
OS: Overall Survival
PPI: Protein-protein Interaction
PTC: Papillary Thyroid Carcinoma
ROC: Receiver Operating Characteristic
siRNAs: small interfering RNAs
SNPs: Single-nucleotide Polymorphisms
TC: Thyroid Cancer
TCGA: The Cancer Genome Atlas
WGCNA: Weighted Gene Co-expression Network Analysis
